# Does Online Search Behavior Coincide with *Candida auris* Cases? An Exploratory Study

**DOI:** 10.3390/jof5020044

**Published:** 2019-06-04

**Authors:** Katja Saris, Jacques F. Meis, Jesús Rodriguez Baño, Evelina Tacconelli, Tom H. van de Belt, Andreas Voss

**Affiliations:** 1 Department of Medical Microbiology and Infectious Diseases, Canisius-Wilhelmina Hospital (CWZ), 6532SZ Nijmegen, The Netherlands; katjasaris@gmail.com (K.S.); jacques.meis@gmail.com (J.F.M.); 2 REshape Center for Innovation, Radboudumc, 6525GA Nijmegen, The Netherlands; tom.vandebelt@radboudumc.nl; 3 Department of Medical Microbiology, Radboudumc, 6525GA Nijmegen, The Netherlands; 4 Center of Expertise in Mycology, Radboudumc/CWZ, 6525GA Nijmegen, The Netherlands; 5 Department of Medicine, Infectious Diseases, Microbiology and Preventive Medicine Unit, Hospital Universitario Virgen Macarena, University of Sevilla, IBiS, 41009 Seville, Spain; jesusrb@us.es; 6 Department of Diagnostic and Public Health, Infectious Diseases, University of Verona, 37129 Verona, Italy; evelina.tacconelli@univr.it; 7 Radboud Institute for Health sciences, Radboudumc, 6525GA Nijmegen, The Netherlands

**Keywords:** *Candida auris*, cases, Google Trends, search behavior, surveillance

## Abstract

*Candida auris* is an emerging multidrug resistant infectious yeast which is challenging to eradicate and despite available laboratory methods is still difficult to identify especially in less developed countries. To limit the rapid spread of *C. auris*, quick and accurate detection is essential. From the perspective of disease surveillance, additional methods of tracking this yeast are needed. In order to increase global preparedness, we explored the use of online search behavior to monitor the recent global spread of *C. auris*. We used Google Trends to assess online search behavior on *C. auris* from January 2016 until August 2018. Weekly Google Trends results were counted as hits and compared to confirmed *C. auris* cases obtained via publications and a global expert network of key opinion leaders. A total of 44 countries generated a hit, of which 30% (13/44) were confirmed known cases, 34% (15/44) were missed known cases, 34% (15/44) were hits for unknown cases, and 2% (1/44) were confirmed unknown cases. Conclusions: Google Trends searches is rapidly able to provide information on countries with an increased search interest in *C. auris*. However, Google Trends search results do not generally coincide with *C. auris* cases or clusters. This study did show that using Google Trends provides both insight into the known and highlights the unknown, providing potential for surveillance and tracking and hence aid in taking timely precautionary measures.

## 1. Introduction

*Candida auris* is an emerging multidrug resistant infectious yeast which is fast spreading globally and has reached all inhabited continents [1]. It predominantly spreads in the nosocomial setting. Since the first report of *C. auris* in 2009 [2], this yeast has caused increasing numbers of infections and outbreaks around the globe [3,4,5,6,7,8,9]. *Candida auris* causes bloodstream infections, wound infections, and otitis [10,11]. It mainly affects vulnerable patient groups such as intensive care patients and neonates treated in health care facilities [12]. Additionally, due to the predominant nosocomial spread of *C. auris*, it is important that health care facilities be aware and ready in case of *C. auris* infection (case) or outbreak (cluster) [10,13]. Outbreaks in Europe and the USA could be related to introductions of *C. auris* into the healthcare environments from one or more of the four known clades [14]. Due to increasing awareness of health care workers, government agencies, and researchers and better biochemical identification methods due to updates in lab system databases, *C. auris* becomes easier to identify [15]. However, this is still not the case in less developed countries. Additionally, the multidrug resistant nature of *C. auris* together with its high virulence [12], its capability to survive from weeks to months in the environment of hospital surfaces, and difficulty in decolonization [7,16] are reasons for grave concern and raised global awareness and alarm [9,17,18,19]. This is so especially as we are still looking for the best way to track the spread of *C. auris* not just globally but also locally within institutions [20,21,22,23]. Therefore, there is a pressing need for accurate, timely, and efficient ways of tracking *C. auris*. 

Since the Internet is widely used, it is an interesting source of information which is often used for academic research such as analysing (micro) blog content or global online search behavior [24]. In particular, the Internet has shown to be a valuable source to detect and monitor disease outbreaks such as the flu and others [25,26]. Online search behavior has also successfully been used to predict other infectious diseases including Methicillin-resistant *Staphylococcus aureus* (MRSA) outbreaks based on search entries [27]. Since *C. auris* cases are often location specific, online search behavior data could be a valuable source of information about emerging *C. auris* cases and clusters. We explored the use of online search behavior to monitor the recent global spread of *C. auris* in order to increase global preparedness. 

## 2. Materials and Methods

### 2.1. Design and Setting

An exploratory global study was conducted in which online search behavior on *Candida auris* was compared to confirmed *C. auris* cases or clusters. The latter were derived from scientific peer reviewed publications of *C. auris* cases and clusters, searches in grey literature, and internationally conducted mini surveys within an expert network of key opinion leaders (KOLs) asking about knowledge of national *C. auris* cases or occurrence of clusters. 

Online search behavior assessment on *C. auris* was performed between 1 January 2016 and 31 July 2018. Key opinion leaders were asked during 2017 and the first half of 2018 to use their network to provide unpublished information on cases or clusters in their country of residence. The KOLs in mycology and/or infection control were selected from those countries that showed activity in their online search behavior for *C. auris* but had no published occurrence of cases or clusters in the literature within the set search interval. 

### 2.2. Identifying C. auris Cases and Clusters

A search for *C. auris* was conducted in the scientific databases PubMed and Web of Science for the years 2016 to 2018. Additionally, references in the literature were checked and, when relevant, added to the literature list. Grey literature was selected based on it being mentioned in the reference list of selected scientific publications. Websites of interest for grey literature were public health websites and conference websites in the field of microbiology and mycology. Literature had to be in English. Literature on scientific evaluations, such as antifungal resistance analyses or typing, and reports based only on previously obtained isolates without cases or clusters of *C. auris* detected between 2016 and the middle of 2018 were excluded. 

Key opinion leaders in the field were identified and consulted via expert contacts in the Dutch field of Infection Control and Mycology. The latter sent messages to KOLs in 12 of 16 countries without publications but with Google Trends activity or a hit, (Table 1) asking about their knowledge on *C. auris* cases or clusters in their country and the month and year of the occurrence between 2016 and the middle of 2018. Additionally, the experts gathered information from KOLs at the 20th Congress of the International Society of Human and Animal Mycology (ISHAM 30 June until 4 July 2018 in The Netherlands). Replies of the KOLs provided data used for this study including any unpublished studies or data on *C. auris*. A report on *C. auris* was included if a KOL reported a cluster, infection, or potential index case within the country in the given timeframe. 

### 2.3. Assessing Online Search Behavior

To assess online search behavior, Google Trends (GT) was used [28]. This tool provides insight into search behavior of all people using Google based on specific searches performed in Google Search. It provides the relative frequency of searches for different countries and regions globally in the specified time interval. We used a similar approach as has been used in studies that successfully detected influenza and MRSA outbreaks using GT [26,27]. Although different search engines are used worldwide, our searches were limited to Google Trends since Google covers approximately 82% of all Internet searches [29]. 

Regarding the searches, only the term *Candida auris* was used since this term is indisputable, clear and comprehensive. No specific settings for GT were applied other than the search region that was set to worldwide. Google Trends presents search interest of topics on a scale (0% to 100%) per day instead of an absolute number thus every set search interval will have a maximum and a minimum even when absolute search numbers are low during this interval (e.g. without any peaks in search behavior) [27]. Since *C. auris* is relatively new, with the first case reported in 2009 [2], and outbreaks have not been reported in every country, search intervals were set to one week, starting on 1 January 2016 when *C. auris* picked up global interest. In addition, as of January 2016, Google Trends improved their system on information gathering [28]. The location (country) with the highest (100%) search interest for the term *Candida auris* per week was gathered and defined as a hit. The hits were added to our database. 

### 2.4. Analysis

A Table was created based on the finding of *C. auris* in the literature/KOL information and the GT hits, see Table 1. In addition, information from the literature/KOL and GT was combined and added to a table in alphabetical order (number of cases, country, and period, see Table S1). Countries with overlap in *C. auris* data collection and GT hit were added to a separate table if information on timeframe including month and year of cases was available. Detection of overlap in timeframe is interesting as potential for case or cluster identification. 

### 2.5. Ethical Approval

Since the anonymous data used in this study was derived from the public social media domain without patient involvement and pre-existing contacts of specialists in the field were used, no medical ethical review was needed in The Netherlands. 

## 3. Results

Between 1 January 2016 and August 2018, we performed searches in GT resulting in hits in 29 countries. Twelve KOLs were consulted, resulting in 1 unknown case in 1 country (Australia).

Table 1 shows the combination of the GT hits with published and unpublished *C. auris* reports within the search period. The first column (Lit+ GT+) shows the countries with published reports on *C. auris* cases or clusters between 2016 and the middle of 2018 with at least one hit in GT during the search period (*n* = 13). More countries had published reports on *C. auris* but showed no hits in the GT search (*n* = 15), which is shown in the second column (Lit+ GT−). The largest group had no published reports on *C. auris* cases or clusters but had at least one hit in the GT search (GT+ Lit−) (*n* = 16). Finally, the KOL information was used to assess whether countries with GT hits but no present literature were indeed without cases. Consequently, the final column shows the true positive countries (GT+, Lit−, KOL+) in light grey (*n* = 1) and false positive countries for *C. auris* cases or clusters (GT+, Lit−, KOL−) in dark grey (*n* = 15).

The combined literature search and GT hit collection, resulted in a total of 44 countries. Of these, 30% (13/44) were identified known *C. auris* cases by GT search, 34% (15/44) were missed known *C. auris* cases by GT search behavior, 2% (1/44) were confirmed unknown *C. auris* cases, and 34% (15/44) were confirmed hits for unknown cases.

Countries with overlap in *C. auris* data collection presented in the literature and GT hit within the set search interval were Colombia, the UK and the USA. All three publications provided a date range for case or cluster collection (see Table 2). 

## 4. Discussion

In this study, we explored the potential value of assessing online search behavior on *Candida auris* in relation to *C. auris* cases or clusters. One third of the hits found in GT were already known *C. auris* cases, well described in the literature. It is encouraging that a freely available, easy-to-access tool such as GT shows these cases. Additionally, our approach rapidly provided us with several new insights into *C. auris* cases, including the finding of one country which indeed had a *C. auris* case not known publicly. Interestingly, one third of the GT hits were not confirmed by official data from publications or KOLs. On the other hand, one third of known *C. auris* cases were not identified via online search behavior.

One third (34%) of the hits were not confirmed by literature or KOLs. However, we cannot conclude that these hits are missed *C. auris* cases, particularly since not all hits were confirmed by KOL reply. This finding suggests that one third of the countries had an increased search interest for *C. auris* within the 1.5 year set-time interval as these countries peaked in GT. This increased interest could be on any subject regarding *C. auris*. However, for a country to come up as a hit in this study, it means that online search interest within this country was higher than in any other nation, suggesting an increased interest in *C. auris* and a potential increase in awareness which is essential for the adequate identification, treatment, and prevention of this yeast pathogen. This finding highlights the potential of using online search behavior such as via GT for governments and researchers as a new way of gathering information, which is strengthened by the knowledge that we live in a digital world in which we can assume that *C. auris* is discussed online. 

We used Google Trends to identify *C. auris* cases as has been done for influenza and MRSA outbreaks previously. However, it is best used for high prevalence, non-emerging but prevalent diseases, which is not yet the case for *C. auris* [26]. *Candida auris* infections are relatively new and the identification, treatment, and prevention measures are not completely understood and applicable worldwide [9]. Therefore *C. auris* might not have the prevalence for proper cluster or case detection via Google Trends. Especially, since many published *C. auris* reports consist of <10 cases [6,30,31,32]. Additionally, the overlap found in published *C. auris* case collection and GT hit was minimal with only three countries out of the 28 countries reported to have *C. auris* cases or clusters (Table 1 and Table 2). Moreover, even for these three countries, the hits in GT currently cannot be linked exclusively to the published case collection. However, it is to be expected that the ‘critical mass’ for *C. auris* detection via Internet will increase as this yeast continues to spread globally, infection cases become more common and well reported, and the potential for using social media and GT in particular becomes more valuable. 

Even though the Internet is indeed worldwide, there is still a difference between more developed countries and developing countries in its use and accessibility [33]. This might have contributed to the low (2%, *n* = 1) number of developed countries (Australia) with an identified unknown case and the higher number of missed known cases (34%) in countries on the African and Asian continents with potentially less internet access. However, in our current fast-changing digital era, it is essential that we explore new ways to track and detect diseases. Using the Internet is one of those methods [24].

Furthermore, as misidentification of *C. auris* is still a problem in many countries [12], *C. auris* might go undetected even when present. Since detection is a predicament in some facilities that are not using up-to-date equipment or standards [34], possible *C. auris* isolates might be sent to foreign laboratories for confirmation. Identification in a facility in another country can possibly result in a skewed peak search interest in the country of detection rather than the country of *C. auris* isolate origin. This might be true, for example, for cases detected in the USA that originated in the Middle East and infections detected in the UK that had an origin in South Africa [9,23]. This potentially contributes to the relatively high percentage of missed known cases (34%) and identified hits for unknown cases (34%) found. Additionally, search activity could have been influenced by publications from national organisations such as the CDC and PHE. However, this study does show that people are searching for *C. auris* and that this yeast is becoming more and more well-known globally. 

## 5. Limitations

The timeframe for the publications to be included was set to either case collection or duration of the study between 2016 and the middle of 2018 or (first) publication from 2016 to the middle of 2018. This timeframe was chosen to coincide with the Google Trends search period. It did include some of the recent major outbreaks, such as known reported outbreaks in the UK and South Africa [7,35]. However, to create Table 1, the timeframe used did not take into account differences in date of the GT hit and publication or KOL information date on *C. auris* within the set timeframe. 

Since GT shows the relative frequency of searches for different countries and regions in a specified time interval, the result differs depending on the time interval chosen. However, since we used search intervals of a week, we believe this represents true search behavior better than looking at monthly GT searches. 

Finally, we only looked at *Candida auris* as search term in Google Trends. This can result in a variety of hits on this topic. In this exploratory study we have specifically looked in the literature and networks for *C. auris* cases and clusters, whereas Google searches conducted with the term *Candida auris* may have been looking for information on resistance or detection of *C. auris* and therefore might give a skewed image for the number of local hits counted as cases or clusters. However, interest in *C. auris* in any form might be an indication of infection or potential cases and at least of increased awareness.

## 6. Conclusions

Google Trends searches rapidly provide information on countries with an increased search interest in *C. auris*. Currently, this freely available, easy-to-access tool is encouraging research by trying to correlate search behavior for *C. auris* in countries with known infections or cases. However, at the moment online search behavior for *C. auris* generates a relatively high misidentification of published cases and hits for unknown cases and shows little overlap in timeframe for case collection and Google Trends search behavior. Therefore, it can be concluded that Google Trends searches currently do not generally coincide with *C. auris* cases or clusters. More research is needed, especially at a time when *C. auris* becomes endemic and thus does not trigger searches by those trying to get informed about an emerging problem. At that point, Google Trends might be better suited to identify peaks in occurrence, e.g., outbreaks, as it does for influenza [25]. Particularly in a fast-changing digital world, health care organisations, professionals, and researchers are prompted to look for new methods of surveillance and earlier detection of diseases. This study shows that using such a tool both provides insight into the known and highlights the unknown, providing potential for surveillance and tracking and hence aid in taking timely precautionary measures.

## Figures and Tables

**Table 1:** Overview of *C. auris* in published literature, Google Trends and key opinion leaders (KOL) from 2016 to the middle of 2018.

Lit+ GT+ (n = 13)	Lit+ GT- (n = 15)	GT+ Lit- (n = 16)
Austria	Bangladesh	Australia
Belgium	China	Belarus *
Canada	France	Brazil
Colombia	Israel	Bulgaria
Germany	Japan	Chile *
India	Kuwait	Croatia
Panama	Malaysia	Finland
Spain	Norway	Ireland
Switzerland	Oman	Italy
UK	Pakistan	Mexico
United Arab Emirates	Russia	The Netherlands
USA	Saudi Arabia	Peru *
Venezuela	Singapore	Philippines *
	South Africa	Poland
	South Korea	Sweden
		Thailand

Light grey shading is KOL positive (*n* = 1), dark grey shading is KOL negative (*n* = 15), * No KOL confirmation was obtained.

**Table 2:** A light grey shading of the overlap in the published *C. auris* cases or clusters and GT hits for 2016 to the middle of 2018.

Country	Timeframe (and Number of Cases)	GT hits
Colombia	February–July 2016 (17) [5]	July 2016, August 2016 (2×), October 2016 (4×), February 2017, July 2017, August 2017, December 2017, March 2018, April 2018, May 2018 (2×)
UK ^a^	2 February 2015–31 August 2017 (70) [23] April 2015–July 2016 (50) [7]	January 2016, March 2016 (3×), April 2016, May 2016, June 2016, August 2016 (2×), December 2016, February 2017 (2×), August 2017, March 2018, April 2018
USA ^a^	May 2013–August 2016 (7) of which 5 in April–August 2016 [30]	June 2016, July 2016, March 2017, April 2017, March 2018 (2×)

^a^ On 1 July 2016, both the CDC and Public Health England (PHE) issued the first notification for *C. auris*.

## Supplementary Materials

The following are available online at https://www.mdpi.com/2309-608X/5/2/44/s1, Table S1: Overview of (un)published *C. auris* outbreaks or cases, KOL information and GT hits 2016-first half 2018.
