# Supplementary material for: Does Online Search Behavior Coincide with Candida auris Cases? An Exploratory Study
Source: J Fungi (Basel). 2019 Jun 4;5(2):44. doi: 10.3390/jof5020044 (PMC6616941; doi:10.3390/jof5020044)
Supplement: Supplementary file 1 [file jof-05-00044-s001.pdf]

Table S1. Overview of (un)published *C. auris* outbreaks or cases, KOL information and GT hits 2016-first half 2018.

**Country**	**Time (number of cases)**	**First (online) Publication date [reference]**	**GT hit**	**KOL confirmation date (number of cases)**
Australia		-	Jan 2017, July 2017, April 2018	7 August 2018 (Victoria) 1 case
Austria	(1)	ECDC (29) March 2018 [1]	September 2016 (2x), November 2016 (3x), May 2018, June 2018	January 2018 (1 Ear isolate in Vienna)
Belarus	-	-	March 2017, May 2017	
Belgium	No indication of date (1)	ECDC (29) March 2018 [1,2]	October 2017, January 2018	September 2017 (1)
Brazil	-	-	May 2016	No reported infection
Bulgaria	-	-	July 2016, June 2017	No reported infection
Canada	May 2017 (1)	July (20) 2017 [3]	August 2016, February 2018	-
Chile	-	-	June 2017, August 2017 (2x)	
China	Between January 2011 and October 2017, retesting of misidentified *C. auris*. (15 cases)<br><br>Date not stated (1)	July 2018 [4]<br><br>May (18) 2018 [5]		
Colombia	February - July 2016 (17)<br><br>3 cases:<br>November 2013+December 2014+February 2015	January (4) 2017 [6]<br><br>Accepted (30) January 2018 [7]	July 2016, August 2016 (2x), October 2016 (4x), February 2017, July 2017, August 2017, December 2017, March 2018, April 2018, May 2018 (2x)	

Croatia	-	-	July 2016, May 2017, July 2018	No reported infection
France	Somewhere between 2013-2017 (2)	March (29) 2018 [1]		
Finland	-	-	January 2016, July 2016, December 2016, May 2018	No reported infection
Germany	November 2015 - August 2017 (5)<br><br>2013-2017 (7)	Publication on website on (5) September 2017 [8]<br><br>March (29) 2018 [1]	September 2017, January 2018 (3x), February 2018, March 2018	-
India	2009-2017 (>350)	February (20) 2017 [9]<br>July (29) 2016 [10]	January 2016, April 2016	
Ireland	-	-	October 2016, September 2017, October 2017, November 2017, January 2018	No reported infection
Israel	May - October 2014, April 2015 (6)	January (22) 2017 [11]		-
Italy			June 2018	
Japan	2017 (date not stated) (1)	February 2018 [12]		
Kuwait	May 2014 - September 2017 (56)	April 2018 [13]		
Malaysia	2017 (date not stated) (1)	January (30) 2018 [14]		-
Mexico	-	-	December 2016, July 2017 (2x)	
The Netherlands	-	-	January 2017, June 2018, July 2018	March 2017 (1), June 2018 (1)
Norway	Date not stated (1)	December (12) 2016 [15]		-
Oman	December 2016 - February	May (24) 2017 [16]		-

	2017 (5)<br><br>August 2016 – January 2017 (2)	 <br>June (25) 2017 [17]		
Pakistan	2015 (2)	December (16) 2016 [18]		
Panama	July - October 2016 (9)	September (25) 2017 [19]	November 2016, March 2017, April 2017 (3x), May 2017 (3), September 2017, November 2017, April 2018	
Peru	-	-	June 2017, December 2017	
Philippines	-	-	November 2016, March 2017	
Poland	-	-	January 2017	No reported infection
Russia	Date not stated (1)	May 2018 ECCMID [20]		
Saudi Arabia	December 2017 – February 2018 (3)	March (20) 2018 [21]		
Singapore	2012 (1) / 2016 (1) / early 2017 (1)	July 2018 [22]		
South Africa	October 2012-November 2016 (861)	November (11) 2018 [23]		-
South Korea	2002-2017 (50 isolates)	April 2018 ECCMID [24]		-
Spain	April - June 2016 (8 isolates/4 patients)<br><br>2013-2017 (388)	January (25) 2017 [25]<br><br><br>March (29) 2018 [1]	December 2016, January 2017, august 2017, November 2017, March 2018, May 2018, July 2018 (2x)	-
Sweden	-	-	November 2017	No reported infection

Switzerland	October 2017 (1)	April (26) 2018 [26]	July 2016, September 2016, June 2017, September 2017, February 2018	-
Thailand	-	-	January 2017	
UK	February 2, 2015 – August 31, 2017 (70)<br><br>April 2015 - July 2016 (50)	October (4) 2018 [27]<br><br>October (3) 2016 [28]	January 2016, March 2016 (3x), April 2016, May 2016, June 2016, August 2016 (2x), December 2016, February 2017 (2x), August 2017, March 2018, April 2018	-
United Arab Emirates	September 2017 (1)	May 2018 [29]	October 2017, December 2017 (2x)	-
USA	May 3013 – August 2016 (7) of which 5 in April - August 2016<br><br>From 2013-continuing (457)	November (29) 2016 [30]<br><br>October (31) 2018 [31]	June 2016, June 2016, March 2017, April 2017, March 2018 (2x)	-
Venezuela	March 2012 – July 2013 (18)	July (21) 2016 [32]	February 2017, October 2017, November 2017	-

References

1. Kohlenberg, A.; Struelens, M.J.; Monnet, D.L.; Plachouras, D.; The Candida Auris Survey Collaborative, G. Candida auris: Epidemiological situation, laboratory capacity and preparedness in european union and european economic area countries, 2013 to 2017. *Euro surveillance : bulletin Europeen sur les maladies transmissibles = European communicable disease bulletin* **2018**, *23*.
2. Dewaele, K.; Frans, J.; Smismans, A.; Ho, E.; Tollens, T.; Lagrou, K. First case of candida auris infection in belgium in a surgical patient from kuwait. *Acta clinica Belgica* **2018**, 1-8.
3. Schwartz, I.; Hammond, G. First reported case of multidrugresistant *candida auris* in canada. *Can Commun Dis Rep.* **2017**, *2017*, 150-153.
4. Tian, S.; Rong, C.; Nian, H.; Li, F.; Chu, Y.; Cheng, S.; Shang, H. First cases and risk factors of super yeast candida auris infection or colonization from shenyang, china. *Emerging microbes & infections* **2018**, *7*, 128.

5. Wang, X.; Bing, J.; Zheng, Q.; Zhang, F.; Liu, J.; Yue, H.; Tao, L.; Du, H.; Wang, Y.; Wang, H.*, et al.* The first isolate of candida auris in china: Clinical and biological aspects. *Emerging microbes & infections* **2018**, *7*, 93.
6. Morales-López, S.E.; Parra-Giraldo, C.M.; Ceballos-Garzón, A.; Martínez, H.P.; Rodríguez, G.J.; Álvarez-Moreno, C.A.; Rodríguez, J.Y. Invasive infections with multidrug-resistant yeast candida auris, colombia. *Emerg Infect Dis* **2017**, *23*, 162-164.
7. Parra-Giraldo, C.M.; Valderrama, S.L.; Cortes-Fraile, G.; Garzon, J.R.; Ariza, B.E.; Morio, F.; Linares-Linares, M.Y.; Ceballos-Garzon, A.; de la Hoz, A.; Hernandez, C.*, et al.* First report of sporadic cases of candida auris in colombia. *International journal of infectious diseases : IJID : official publication of the International Society for Infectious Diseases* **2018**, *69*, 63-67.
8. Nationales Referenzzentrum für invasive Pilzinfektionen. *Infektionen durch candida auris – stellungnahme des nrzmyk jena.*; Jena: NRZMyk, 2016.
9. Rudramurthy, S.M.; Chakrabarti, A.; Paul, R.A.; Sood, P.; Kaur, H.; Capoor, M.R.; Kindo, A.J.; Marak, R.S.K.; Arora, A.; Sardana, R.*, et al.* Candida auris candidaemia in indian icus: Analysis of risk factors. *The Journal of antimicrobial chemotherapy* **2017**, *72*, 1794-1801.
10. Sharma, C.; Kumar, N.; Pandey, R.; Meis, J.F.; Chowdhary, A. Whole genome sequencing of emerging multidrug resistant candida auris isolates in india demonstrates low genetic variation. *New Microbes New Infect* **2016**, *13*, 77-82.
11. Ben-Ami, R.; Berman, J.; Novikov, A.; Bash, E.; Shachor-Meyouhas, Y.; Zakin, S.; Maor, Y.; Tarabia, J.; Schechner, V.; Adler, A.*, et al.* Multidrug-resistant candida haemulonii and c. Auris, tel aviv, israel. *Emerg Infect Dis* **2017**, *23*, 195-203.
12. Iguchi, S.; Mizushima, R.; Kamada, K.; Itakura, Y.; Yoshida, A.; Uzawa, Y.; Arai, Y.; Takaoka, M.; Sato, S.; Goto, A.*, et al.* The second candida auris isolate from aural discharge in japan. *Japanese journal of infectious diseases* **2018**, *71*, 174-175.
13. Khan, Z.; Ahmad, S.; Al-Sweih, N.; Joseph, L.; Alfouzan, W.; Asadzadeh, M. Increasing prevalence, molecular characterization and antifungal drug susceptibility of serial candida auris isolates in kuwait. *PloS one* **2018**, *13*, e0195743.
14. Mohd Tap, R.; Lim, T.C.; Kamarudin, N.A.; Ginsapu, S.J.; Abd Razak, M.F.; Ahmad, N.; Amran, F. A fatal case of candida auris and candida tropicalis candidemia in neutropenic patient. *Mycopathologia* **2018**, *183*, 559-564.
15. European Centre for Disease Prevention and Control (ECDC). Candida auris in healthcare settings –europe. ECDC, Ed. Stockholm: ECDC, 2016.
16. Al-Siyabi, T.; Al Busaidi, I.; Balkhair, A.; Al-Muharrmi, Z.; Al-Salti, M.; Al'Adawi, B. First report of candida auris in oman: Clinical and microbiological description of five candidemia cases. *J Infect* **2017**, *75*, 373-376.
17. Mohsin, J.; Hagen, F.; Al-Balushi, Z.A.M.; de Hoog, G.S.; Chowdhary, A.; Meis, J.F.; Al-Hatmi, A.M.S. The first cases of candida auris candidaemia in oman. *Mycoses* **2017**, *60*, 569-575.
18. Lockhart, S.R.; Etienne, K.A.; Vallabhaneni, S.; Farooqi, J.; Chowdhary, A.; Govender, N.P.; Colombo, A.L.; Calvo, B.; Cuomo, C.A.; Desjardins, C.A.*, et al.* Simultaneous emergence of multidrug-resistant candida auris on 3 continents confirmed by whole-genome sequencing and epidemiological analyses. *Clin Infect Dis* **2017**, *64*, 134-140.
19. Arauz, A.B.; Caceres, D.H.; Santiago, E.; Armstrong, P.; Arosemena, S.; Ramos, C.; Espinosa-Bode, A.; Borace, J.; Hayer, L.; Cedeno, I.*, et al.* Isolation of candida auris from 9 patients in central america: Importance of accurate diagnosis and susceptibility testing. *Mycoses* **2018**, *61*, 44-47.
20. Natalya Vasilyeva; Alexander Kruglov; Ivan Mikhailovich Pchelin; Igor Riabinin; Ekaterina Raush; Galina Chilina; Tatiyana Bogomolova; Ilya Bosak; Olga Shurpitskaya; Nikolai Klimko*, et al.* In *P0311 the first russian case of candidaemia due to candida auris*, European Congress of Clinical Microbiology and Infectious Diseases (ECCMID). Madrid, Spain, 2018; Madrid, Spain.
21. Abdalhamid, B.; Almaghrabi, R.; Althawadi, S.; Omrani, A. First report of *candida auris* infections from saudi arabia. *J Infect Public Health* **2018**, *11*, 598-599.

22. Tan, Y.E.; Tan, A.L. Arrival of candida auris fungus in singapore: Report of the first 3 cases. *Annals of the Academy of Medicine, Singapore* **2018**, *47*, 260-262.
23. Govender, N.P.; Magobo, R.E.; Mpembe, R.; Mhlanga, M.; Matlapeng, P.; Corcoran, C.; Govind, C.; Lowman, W.; Senekal, M.; Thomas, J. Candida auris in south africa, 2012-2016. *Emerg Infect Dis* **2018**, *24*, 2036-2040.
24. Yong Jun Kwon; Jong Hee Shin; Eun Jeong Won; Seung A Byun; Min Ji Choi; Seung Jung Kee; Soo Hyun Kim; Myung Geun Shin; Suh, S.P. In *O0527 candida auris isolates from 11 university hospitals in korea: Identification, antifungal susceptibility and multilocus sequence typing*, European Congress of Clinical Microbiology and Infectious Diseases (ECCMID). Madrid, Spain, 2018; Madrid, Spain.
25. Ruiz Gaitan, A.C.; Moret, A.; Lopez Hontangas, J.L.; Molina, J.M.; Aleixandre Lopez, A.I.; Cabezas, A.H.; Mollar Maseres, J.; Arcas, R.C.; Gomez Ruiz, M.D.; Chiveli, M.A.*, et al.* Nosocomial fungemia by candida auris: First four reported cases in continental europe. *Rev Iberoam Micol* **2017**, *34*, 23-27.
26. Riat, A.; Neofytos, D.; Coste, A.; Harbarth, S.; Bizzini, A.; Grandbastien, B.; Pugin, J.; Lamoth, F. First case of candida auris in switzerland: Discussion about preventive strategies. *Swiss Med Wkly* **2018**, *148*, w14622.
27. Eyre, D.W.; Sheppard, A.E.; Madder, H.; Moir, I.; Moroney, R.; Quan, T.P.; Griffiths, D.; George, S.; Butcher, L.; Morgan, M.*, et al.* A candida auris outbreak and its control in an intensive care setting. *N Engl J Med* **2018**, *379*, 1322-1331.
28. Schelenz, S.; Hagen, F.; Rhodes, J.L.; Abdolrasouli, A.; Chowdhary, A.; Hall, A.; Ryan, L.; Shackleton, J.; Trimlett, R.; Meis, J.F.*, et al.* First hospital outbreak of the globally emerging candida auris in a european hospital. *Antimicrob Resist Infect Control* **2016**, *5*, 35.
29. Alatoom, A.; Sartawi, M.; Lawlor, K.; AbdelWareth, L.; Thomsen, J.; Nusair, A.; Mirza, I. Persistent candidemia despite appropriate fungal therapy: First case of candida auris from the united arab emirates. *International journal of infectious diseases : IJID : official publication of the International Society for Infectious Diseases* **2018**, *70*, 36-37.
30. Vallabhaneni, S.; Kallen, A.; Tsay, S.; Chow, N.; Welsh, R.; Kerins, J.; Kemble, S.K.; Pacilli, M.; Black, S.R.; Landon, E.*, et al.* Investigation of the first seven reported cases of candida auris, a globally emerging invasive, multidrug-resistant fungus-united states, may 2013-august 2016. *MMWR. Morb Mortal Wkly Rep.* **2017**, *65*, 1234-1237.
31. Centers for Disease Control and Prevention (CDC). Tracking c. Auris. (Accessed 18 December 2018),
32. Calvo, B.; Melo, A.S.; Perozo-Mena, A.; Hernandez, M.; Francisco, E.C.; Hagen, F.; Meis, J.F.; Colombo, A.L. First report of *candida auris* in america: Clinical and microbiological aspects of 18 episodes of candidemia. *J Infect* **2016**, *73*, 369-374.
